# A study of the responsiveness of the Six-Spot Step Test in people with multiple sclerosis

**DOI:** 10.1007/s13760-025-02887-9

**Published:** 2025-09-04

**Authors:** Esben Køhler, Jacob Callsen, John Kodal Brincks

**Affiliations:** Research Centre for Care and Rehabilitation, Research Program for Balance and Falls, VIA University College, Hedeager 2, 8200 Aarhus N, Denmark

**Keywords:** The Six-Spot Step Test, Functional mobility, Responsiveness, Multiple sclerosis

## Abstract

**Aims:**

This study aimed to evaluate the responsiveness of the Six-Spot Step Test (SSST) in people with multiple sclerosis to assess its capability as a measure for detecting changes in gait and balance capacity following a 10-week training intervention.

**Methods:**

The SSST, Timed 25-Foot Walk, and mini-BESTest were administered to 71 individuals with MS, ranging from mild to severe disability, before and after a 10-week program of progressive resistance and balance training. However, 16 patients were lost to follow-up. This study adhered to the COSMIN framework for reporting and evaluating the psychometric properties of health-related outcome measures.

**Results:**

Spearman's analyses revealed a moderate negative correlation between changes in the SSST and the mini-BESTest (r_s_ = -0.33, *p* = 0.02) and changes in the SSST and the Timed 25-Foot Walk (r_s_ = -0.37, *p* = 0.01). Significant median (min;max) changes and corresponding effect sizes (ES) were observed in the SSST (-1.4 (-11.4;4.7), *p* < 0.001, ES = -0.84), the Mini-BESTest (3 (-3;13), *p* < 0.001, ES = 0.89), and the Timed 25-Foot Walk (0.09 (-0.21;0.54), *p* < 0.001, ES = 0.71).

**Conclusion:**

The SSST serves as a sensitive measure for changes in gait and balance capacity. Furthermore, the consistently large effect sizes observed across all three gait and balance assessments following the exercise intervention suggest that these measures reflect a shared underlying construct—functional mobility—which is essential for independent community living.

## Introduction

Multiple sclerosis (MS) is an autoimmune neurological disorder of the central nervous system, which affects over 2.3 million people—approximately twice as common in women than in men [1]. MS can lead to lesions in the brain, spinal cord, or optic nerves, which causes a variety of symptoms, such as weakness, spasticity, fatigue, and changes in sensation, coordination, vision, and cognition. As a result, it is not surprising that MS negatively affects balance, gait function, and the risk of falling [2].

The Six-Spot Step Test (SSST) is a measure of ambulation that captures complex elements of both gait function and dynamic balance [3]. Additionally, the SSST measures more complex motor tasks than other commonly used tests of gait and functional mobility (e.g., the timed 25-foot walk (T25FW) and the timed “up and go” test (TUG)) [4]. Fay Horak's theoretical framework of balance, which encompasses motor, sensory, and cognitive components, is commonly used to define the balance control system [5]. In this context, the SSST includes fewer balance components compared to more comprehensive tests of balance capacity, such as the mini-Balance Evaluation Systems Test (mini-BESTest) [6, 7]. However, the SSST is more time efficient than this more extensive measures of balance (i.e., mini-BESTest) and requires minimal equipment, space, and assessor training. These qualities make the SSST a convenient and easily implemented tool in clinical settings.

A recent systematic review of the psychometric properties of the SSST revealed that robust evidence supports the reliability and validity of assessing dynamic balance in older adults and people with neurological diseases, including those with Parkinson's disease, stroke, chronic inflammatory polyneuropathy, and MS [8]. Furthermore, studies examining the SSST's validity found a strong to very strong correlation between the SSST and the T25FW and between the SSST and the TUG in people with MS [9, 10]. Similar results have been found in other neurological diseases and older adults with balance problems [11–14]. Only a few studies have investigated the correlation between the SSST and measures of balance capacity (e.g., mini-BESTest). Brincks et al. [11] and Brincks and Callesen [12] found a moderate correlation (−0.64 and −0.62, respectively) between the SSST and the mini-BESTest in both ambulatory people with Parkinson's disease and older adults with self-reported balance problems. However, the systematic review by Aakrann and Brincks [8] of the SSST's psychometric properties did not yield sufficient evidence concerning its longitudinal validity (responsiveness, i.e., the ability of the test to detect meaningful change over time in the construct to be measured), as only two studies of low methodological quality assessed the SSST's responsiveness to intervention-induced changes. In addition, previous studies have only investigated the responsiveness of the SSST against walking-based outcomes (i.e., Multiple Sclerosis Walking Scale-12, the 2-min timed walk, and the T25FW) [15, 16]. Hence, no previous study has evaluated the responsiveness of the SSST after a structured exercise intervention against measures of balance capacity (mini-BESTest) in people with MS. Examining the responsiveness of health-related outcome measures, such as the Six-Spot Step Test (SSST), is essential, as it reflects a measure’s capability to detect meaningful changes over time; an aspect not addressed by construct validity, which only captures relationships between variables at a single point in time.

Studies have found a positive effect of balance training when evaluated by the mini-BESTest and the T25FW [17]. The magnitude of change after balance training in people with MS ranges from approximately 5% to 25% and from non-significant to approximately 10% for the mini-BESTest and the T25FW, respectively [18–22]. Regarding resistance training, the magnitude of change after an intervention is approximately 10% for T25FW [23], while the change in the mini-BESTest is unknown.

This study's primary objective was to examine the responsiveness of the SSST in people with MS after a 10-week intervention period involving resistance and balance control training, using the T25FW and the mini-BESTest as external criteria for gait speed and balance capacity, respectively. It was hypothesized that 1) changes in the SSST were moderately negatively correlated to changes in the mini-BESTest and the T25FW, representing a correlation coefficient between −0.3 and −0.5 in accordance with COSMIN guidelines [24], and 2) a 10-week program involving resistance training and balance control exercises would result in an approximate 15% improvement in the SSST. This anticipated magnitude of improvement aligns with changes observed in the T25FW and the mini-BESTest after undergoing similar exercise treatments with known efficacy on the construct of interest (balance capacity and gait speed).

## Methods

### Participants

All individuals were informed about the study procedures and provided written informed consent following the Declaration of Helsinki. The study was registered at www.clinicaltrials.gov (NCT 02870023) and approved by the Regional Ethics Committee (ID no. 1–10-72–316-15). Participants were included from September 2016 to October 2018 via seven MS clinics across Jutland, Denmark, and were considered eligible for enrolment in the study if the following criteria were met: Adults (≥ 18 years of age) with a confirmed diagnosis of MS phenotypes relapsing–remitting (RR), secondary-progressive (SP), or Primary-progressive (PP), Expanded Disability Status Scale (EDSS): 2.0–6.5 [25], able to walk 100 m, a SSST score > 8 s, a T25FW > 5 s, relapse-free and no adjustment of disease-modifying medication or medication that affects gait performance and spasticity within the past 8 weeks. Participants were excluded from the study if the following criteria were met: Co-morbidity in terms of cognitive disorders or alcohol abuse (based on clinical judgment), pathologies that impaired the participant’s ability to engage in systematic progressive resistance training (PRT) more than once per week, and systematic intensive rehabilitation/training within the last three months.

This study was a post-hoc analysis of a previously published multi-center randomized controlled trial on PRT, balance and motor control training (BMCT), and a waitlist control group in people with MS [26, 27]. The present article followed the consensus standards of the selection of health status measurement instruments (COSMIN) framework [28].

### Intervention

Groups of 3–6 participants were randomly assigned to BMCT, PRT, or a waitlist control group, which received a combination of BMCT and PRT after the waiting period [26]. BMCT and PRT training sessions were conducted twice a week, each lasting approximately one hour, for an intervention period of ten weeks. All PRT exercises were performed on machines, sitting, lying, or standing, and adequately supported. The BMCT training program was based on previously published programs [29–32] and consisted of task-oriented exercises focusing on sitting/sit-to-stand, standing, stepping, walking, and eye-movement training. The waitlist control group waited for ten weeks, maintaining their usual level of care and physical activity. Afterward, they received an intervention with one weekly session of BMCT and one weekly session of PRT. Only data from the period in which the control group was assigned to an exercise intervention were included in the present study. Despite differences in training protocol across groups, the number of sessions and duration were the same.

### Outcome measures

Participants'walking speed (T25FW), complex walking (SSST), and balance capacity (mini-BESTest) were assessed before and after the interventions.

The T25FW [33] and the SSST [3] outcomes were conducted per the original descriptions. For the T25FW, participants obtained the fastest safe walking speed on a 10 m walkway. Participants completed the test two times; the fastest time was used for further analysis. Data from the T25FW was converted to walking speed (m/s). For the SSST, participants were instructed to obtain the fastest safe walking speed across a rectangular course (1 × 5 m) that consisted of six circles, two sidelines with two circles on each sideline, and two baselines with one circle centered on each. Each circle contained a wooden block with a diameter of eight cm and a height of four cm, except the one indicating the starting point. Participants began with their feet in the middle of the empty baseline circle and walked in a criss-cross pattern, shoving the blocks out of the circle with the same leg, alternating between the medial and lateral sides of the foot. Participants completed the test four times, two runs for each leg, starting with two runs for the dominant leg. The average time of the tests was used for further analysis.

The mini-BESTest is a 14-item test that measures balance capacity [6]. The mini-BESTest was scored on a scale of 0 to 28 (a high score equals high balance capacity), subdivided into four divisions: anticipatory transition (score 0–6), postural responses (score 0–6), sensory orientation (score 0–6), and dynamic gait (score 0–10). Thus, the mini-BESTest includes different elements of balance in accordance with Horak's theoretical framework for balance control (i.e., motor, sensory, and cognitive components) [5].

### Data analysis

For the purposes of this responsiveness study, baseline and follow-up scores from all three intervention modes were combined. Regardless of minor differences in effects across training regimens, this approach was justified by the fact that all interventions demonstrated a positive effect on the three outcomes (construct of interest): The T25FW, the SSST, and the mini-BESTest [24]. The effect of BMCT, PRT, and combined BMCT + PRT was on SSST (seconds: 2.6, 0.9, and 1.9, respectively), T25FW (m/s: 0.14, 0.06, and 0.13, respectively), and the mini-BESTest (points: 4.1, 2.1, and 3.7) [26].

A construct approach for testing responsiveness for a performance-based outcome measure was used, and a priori hypotheses, stated in the study aim, were systematically evaluated [24, 28]. Two construct approaches for assessing responsiveness were used: 1) Hypotheses about the expected direction and magnitude of correlations between change in scores of interest were analysed with the Spearman's correlation (r_s_) for evaluating the association among the changes in SSST and T25FW and the mini-BESTest, and 2) hypotheses regarding expected score changes following a treatment with established efficacy were tested using Wilcoxon signed-rank analysis, and the Rank-Biserial method to calculate the effect size with 95% confidence intervals. As the data did not meet the assumptions for normality, evaluations using the Shapiro–Wilk Test, non-parametric analyses were adopted.

Only participants who completed the follow-up assessments were included in the analysis. Accordingly, a per-protocol approach was prioritized over an intention-to-treat strategy, as the primary objective of this study was to assess the responsiveness of the SSST on the construct of interest, which was believed to be more optimally captured following completion of the full intervention period. In light of this analytic approach, to assess attrition bias, a drop-out analysis was performed using baseline data and demographic characteristics of participants lost to follow-up. Baseline data and demographics were described using the median, minimum, maximum, mean, and standard deviation, where applicable. Statistical analyses were conducted using JASP software (version 0.95). A sample size of 50 participants has been considered sufficient for evaluating responsiveness [34].

## Results

### Baseline characteristics

**Fig. 1:**
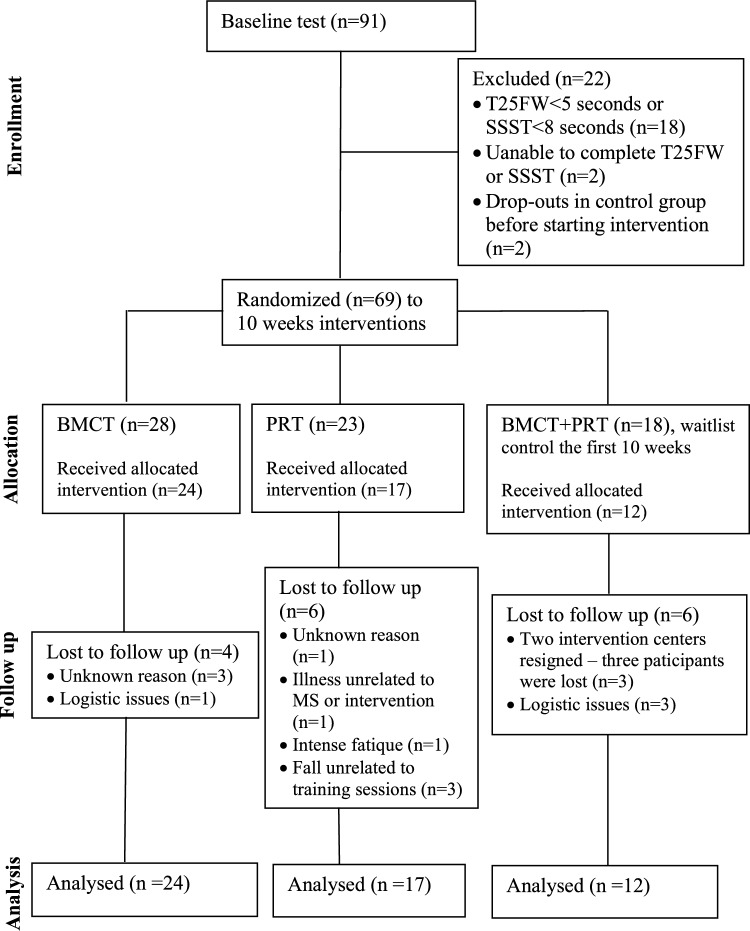
Flow diagram

A total of 91 participants were assessed for eligibility in the original study, and 69 participants were ultimately enrolled (Fig. 1). A total of 16 (23%) participants were lost to follow-up, leaving 53 subjects for the present study. In PRT, 6 out of 23 participants were lost to follow-up (unknown reason n = 1, illness unrelated to MS or intervention n = 1, intensive fatigue n = 1, fall unrelated to training sessions n = 3). In BMCT, 4 out of 28 participants were lost to follow-up (unknown reason n = 3, logistic issues n = 1). In PRT + BMCT, 6 out of 18 were lost to follow-up (logistic reasons n = 3, two centers got below minimum size and resigned n = 3). When comparing the drop-outs to the completers, no differences were seen (Table 1). Of note, one participant presented with an outlying BMI of 51.7 kg/m^2^. However, aside from a notably larger change score in the SSST (11.4 s), no systematic deviations were observed in the mini-BESTest (change score = 0) or the T25FW (change score = 0.12 m/s) compared to the overall sample. Demographic participant characteristics are shown in Table 1.

**Table 1:** Participant characteristics and functional performance at baseline

	Participants at baseline Total (n = 53)	Participants lost to follow-up Total (n = 16)
Sex, F:M	42:11	11:5
Age (years), median (min, max)	52 (38, 75)	52.5 (30, 69)
BMI (kg/m^2^), median (min, max)	24.1 (16.3, 51.7)	26.2 (20.7, 38.8)
EDSS (0–10), median (min, max)	4.0 (2, 6.5)	3.0 (2, 6)
Phenotype RR/SP/PP, n (%)	35 (66)/10 (19)/8 (15)	13 (81)/2 (13)/1 (6)
Gait assistive devices, n (%)	7 (13)	2 (13)
SSST (s), median (min, max)	11.1 (6.9, 40.6)	9.4 (6.4, 18)
Mini-BESTest (0 to 28), median (min, max)	16 (5, 27)	18.5 (3, 27)
T25FW (m/s), median (min, max)	1.26 (0.39, 1.81)	1.36 (0.63,1.91)

SD, Standard Deviation; BMI, Body Mass Index; EDSS, Expanded Disability Status Scale; RR, relapse remitting; SP, secondary Progressive; PP, primary progressive; SSST, Six Spot Step Test; T25FW, Timed 25-Foot Walk; Mini-BESTest, Minimal Balance Evaluation Systems Test

### Responsiveness

Outcome measures before and after the ten-week intervention are shown in Table 2. A significant median difference (min;max) was observed from pre- to post-intervention in all three measures including substantial effect sizes (ES): the SSST (−1.4 (−11.4; 4.7), *p* < 0.001, ES = −0.84), the mini-BESTest (3 (−3;13), *p* < 0.001, ES = 0.89), and the T25FW (0.09 (−0.21;0.54), *p* < 0.001, ES = 0.71).

**Table 2:** Results from before and after 10 weeks of intervention

Outcome measures	Pre (n = 53): Median (min, max)	Post (n = 53): Median (min, max)	Comparison of change from pre to post			
			Median change (min, max)	Change (%)	*p-*value	Effect size (CI)
SSST^a^ (s)	11.1 (6.9, 40.6)	9.3 (5.9, 34.5)	−1.4 (−11.4, 4.7)	−12.6%	< 0.001	−0.84 (−0.91, −0.73)
Mini-BESTest^b^ (0 to 28)	16 (5, 27)	20 (3, 28)	3 (−3, 13)	18.8%	< 0.001	0.89 (0.81, 0.95)
T25FW ^b^ (m/s)	1.26 (0.39, 1.81)	1.43 (0.28, 2.24)	0.09 (−0.21, 0.54)	7.2%	< 0.001	0.71 (0.53, 0.83)

SSST, Six Spot Step Test; T25FW, Timed 25-Foot Walk; Mini-BESTest, Minimal Balance Evaluation Systems Test; CI, Confidence interval

^a^ Decrease reflects improved function

^b^ Increase reflects improved function

**Fig. 2:**
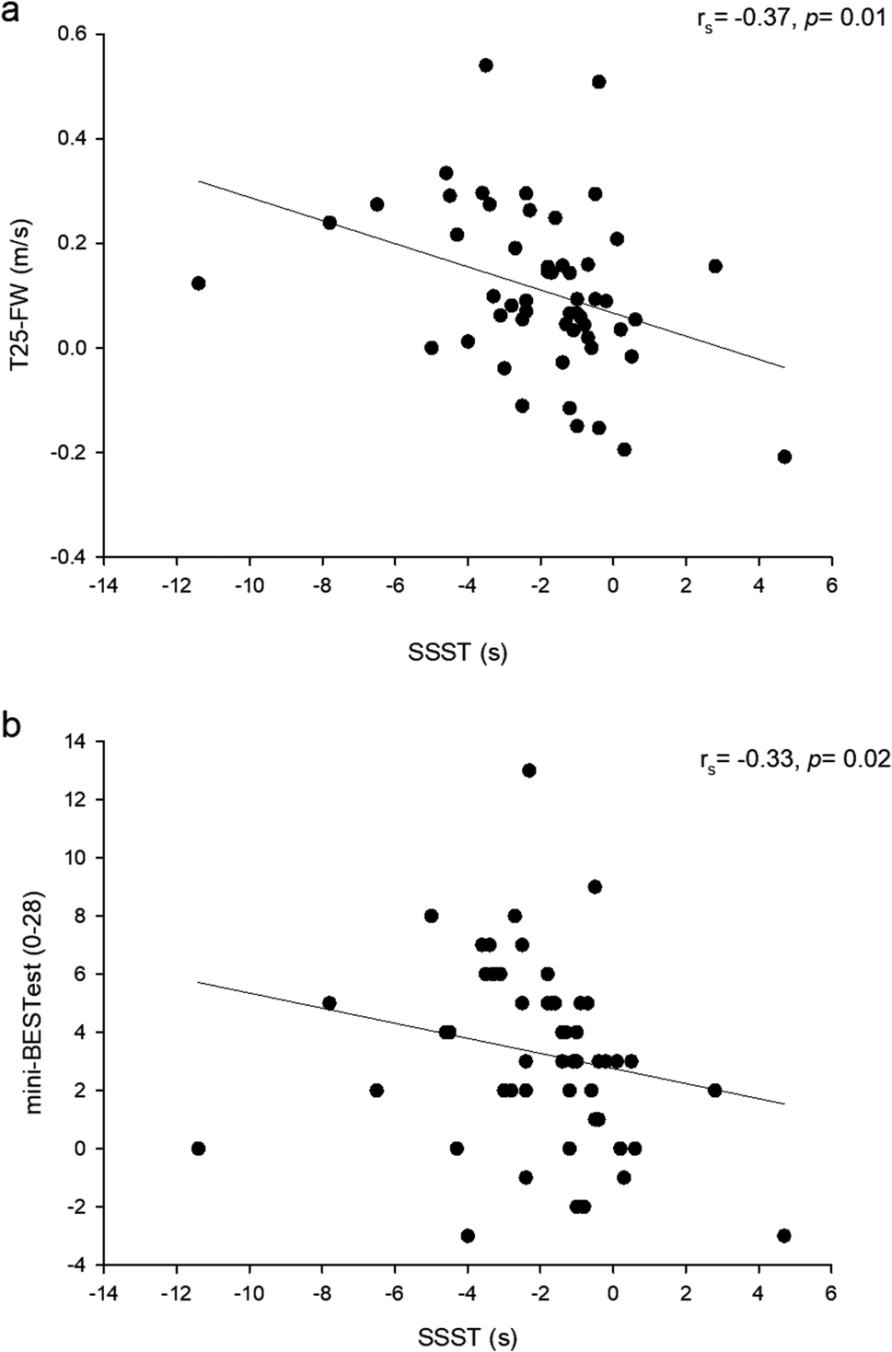
Scatterplot showing relations between the change from pre to post in time of the Six-Spot Step Test (SSST) and the mini-BESTest (**a**, r_s_ = −0.33 (95% CI:−0.55; −0.07), *p* = 0.02) and the timed 25-foot walk (T25FW) (**b**, r_s_ = −0.37 (95% CI: −0.58; −0.11), *p* = 0.01)

The Spearman's analyses found a moderate negative correlation between changes in the SSST and the mini-BESTest (r_s_ = −0.33, *p* = 0.02) (Fig. 2a). A moderate negative correlation was also found between the changes in SSST and the T25FW (r_s_ = −0.37, *p* = 0.01) (Fig. 2b). Additionally, the correlations between changes in the mini-BESTest subdomains and the SSST ranged from weak to moderate. Specifically, weak correlations were observed for sensory orientation, postural responses, and dynamic gait (r_s_ = −0.04, *p* = 0.75; r_s_ = −0.08, *p* = 0.58; r_s_ = −0.24, *p* = 0.08, respectively), while a moderate and significant correlation was found in anticipatory postural adjustments (r_s_ = −0.39, *p* = 0.004).

## Discussion

To the best of our knowledge, this study is the first to investigate the responsiveness of the SSST concerning balance capacity, as measured by the mini-BESTest. Previous studies have only examined the responsiveness between the SSST to gait speed or straight-line walking [15, 16]. Furthermore, including a clearly defined hypothesis regarding the expected relationships between a commonly used gait measure in MS rehabilitation (T25FW) and balance capacity enhances the evaluation of the SSST's psychometric properties, particularly its responsiveness, which has been insufficiently explored. As expected, a negative moderate correlation was found between the changes in the SSST and the changes in both the mini-BESTest (r_s_ = −0.33) and the T25FW (r_s_ = −0.37) after 10 weeks of combined resistance and balance control training in people with MS. Additionally, a notable improvement of 12.6% was found in the SSST, compared to 7.2% in the T25FW and 18.8% in the mini-BESTest.

The mini-BESTest is a widely recognized and extensively validated tool for assessing balance capacity [35], making it an appropriate choice for assessing the responsiveness of the SSST. The mini-BESTest includes tasks similar to those in the SSST, such as standing on one leg, adjusting gait speed, walking with pivot turns, and assessing ambulatory abilities. These similarities coincide with the findings of our subanalysis, which demonstrated a moderate correlation between changes in specific subdomains of the mini-BESTest, namely, dynamic gait and anticipatory postural adjustments, and changes in the SSST (r_s_ = −0.39 and −0.24, respectively). Other tasks bear little resemblance to the SSST, including compensatory stepping corrections in forward, backward, and lateral directions, standing on a firm surface, a foam surface, or an incline ramp, and walking with head turns. This aligns with our correlation analyses, which examined the relationship between changes in the subdomains, sensory orientation, postural responses (reactive strategies), and improvements in the SSST, yielding correlation coefficients of r_s_ = −0.04 and −0.08, respectively. Thus, it is plausible that the multifaceted nature of the mini-BESTest, where only a portion of tasks resembles the SSST, could explain why a moderate, rather than a strong, correlation was found between changes in the SSST and the mini-BESTest.

The SSST is a measure that largely consists of gait. However, only a moderate correlation was found between the SSST and a commonly used measure of gait (T25FW). The responsiveness of the SSST and the T25FW found in the present study is in accordance with previous studies [15, 16]. A possible explanation could be found in the content of the SSST itself. While the SSST might seem like a simple test of gait, it is a much more complex motor task than the T25FW, which requires the ability to change direction and walking speed, walking agility, and balancing on one leg while simultaneously gently shoving blocks, none of which are found in the T25FW. It is, therefore, believed that the much more complex balance requirements of the SSST are the main reason for not finding a stronger correlation between the SSST and T25FW.

A 10-week program combining resistance training with balance exercises led to a 12.6% improvement in the SSST. Additionally, improvements of 7.2% in the T25FW and 18.8% in the mini-BESTest were observed. While there were some differences in the magnitude of change between the SSST, the T25FW, and the mini-BESTest, this was in accordance with the hypothesis. The magnitude of change for both the T25FW and the mini-BESTest found in the present study followed the changes observed in previous studies after resistance and/or balance training in people with MS [1, 18–22]. The fact that the greatest improvement was found in the measure of balance capacity (i.e., mini-BESTest) is, therefore, not surprising, as the intervention consisted of resistance and balance control training —an intervention specifically aimed at improving balance. However, changes were reasonably similar across the SSST, the T25FW, and the mini-BESTest, suggesting that the responsiveness of the measures is quite comparable. Moreover, the effect sizes found after the 10-week intervention were also fairly similar across the SSST, the T25FW, and the mini-BESTest (i.e., 0.84, 0.89, and 0.71, respectively).80. 19, 2

The results of the present study inform about the psychometric properties of the SSST and provide necessary information on the responsiveness after commonly used training interventions. With the convenience, time efficiency, and implementation in clinical settings of the SSST, one could use the SSST to evaluate functional mobility after training interventions for people with MS in clinical practice. Furthermore, a recently conducted systematic review of the SSST, based on COSMIN guidelines, concluded that the SSST is a valid and reliable tool for individuals with Parkinson’s disease, multiple sclerosis, stroke, chronic inflammatory polyneuropathy, and older adults with perceived balance impairments [8]. Although differences exist in diagnoses and symptoms, similarities in gait and balance impairments suggest that the SSST's responsiveness may extend to these populations, further enhancing the clinical applicability of this study’s findings. However, further research is necessary to validate this hypothesis.

When interpreting the study's results, some potential limitations should be considered. First, although comparing baseline data between participants and drops-outs indicated no differences between groups, the substantial drop-out rate (i.e., 23%) increases the risk of attrition bias. Second, the SSST was only compared to gait measures with limited balance requirements (i.e., T25FW). Other commonly used gait measures, such as the TUG, involve more complex balance demands, although not as intricate as those required by the SSST. In this context, the TUG could be an appropriate gait measure. Third, the correlations between the subdomains of the Mini-BESTest and the SSST should be interpreted cautiously, as the subdomain scores of the Mini-BESTest have not been formally validated as distinct measures of balance control [36]. Fourth, responsiveness and the Minimal Clinically Important Difference (MCID) or Minimal Important Difference (MID) are often examined together conceptually for a more comprehensive insight into an outcome measure´s psychometric properties, typically through distribution-based and anchor-based approaches. Responsiveness represents an instrument’s ability to detect changes, whereas the MID/MCID denotes the smallest score or change in score that would likely be important from a patient’s or clinician’s perspective. Importantly, the findings of this study are limited to the responsiveness of the SSST, and further research is required to determine the MID/MCID for the SSST.

## Conclusion

In conclusion, in a sample of people with MS exhibiting mild to severe disability, moderate correlations were observed between changes in the SSST and changes in the mini-BESTest and the T25FW, respectively. Furthermore, comparable effect sizes were found among these three gait and balance measures, suggesting similar responses to a combined strength and balance exercise intervention. Given that the SSST assesses both straightforward and complex dynamic walking tasks, these findings align with expectations and underscore the SSST's longitudinal validity as a responsive measure of functional mobility.

## Data Availability

The data that support the findings of this study are not openly available due to reasons of sensitivity and are available from the corresponding author upon reasonable request. Data are located in controlled access data storage at VIA University College.

## References

[1] Browne P, Chandraratna D, Angood C, Tremlett H, Baker C, Taylor BV, Thompson AJ (2014) Atlas of Multiple Sclerosis 2013: a growing global problem with widespread inequity. Neur 83(11):1022–1024. 10.1212/WNL.0000000000000768 10.1212/WNL.0000000000000768 PMC4162299 25200713

[2] Cameron MH, Nilsagard Y (2018) Balance, gait, and falls in multiple sclerosis. Handb Clini Neur 159:237–250. 10.1016/B978-0-444-63916-5.00015-X 10.1016/B978-0-444-63916-5.00015-X 30482317

[3] Nieuwenhuis MM, Van Tongeren H, Sørensen PS, Ravnborg M (2006) The six spot step test: a new measurement for walking ability in multiple sclerosis. Mult Scler J 12:495–500. 10.1191/1352458506ms1293oa 10.1191/1352458506ms1293oa 16900764

[4] Fritz NE, Jiang A, Keller J, Zackowski KM (2016) Utility of the Six-Spot Step Test as a measure of walking performance in ambulatory individuals with multiple sclerosis. Arch Phy Med Rehab 97:507–512. 10.1016/j.apmr.2015.10.100 10.1016/j.apmr.2015.10.100 26577146

[5] Horak FB (2006) Postural orientation and equilibrium: what do we need to know about neural control of balance to prevent falls? Age Ageing 35 Suppl 2: ii7-ii11. 10.1093/ageing/afl077 10.1093/ageing/afl077 16926210

[6] Franchignoni F, Horak F, Godi M, Nardone A, Giordano A (2010) Using psychometric techniques to improve the Balance Evaluation Systems Test: the mini-BESTest. J Rehabil Med 42:323–331. 10.2340/16501977-0537 20461334 10.2340/16501977-0537 PMC3228839

[7] Di Carlo S, Bravini E, Vercelli S, Massazza G, Ferriero G (2016) The Mini-BESTest: a review of psychometric properties. Int J Rehabil Res 39:97–105. 10.1097/MRR.0000000000000153 26795715 10.1097/MRR.0000000000000153

[8] Aakrann EB, Brincks J (2024) The psychometric properties of the Six-Spot Step Test - a systematic review using the COSMIN guidelines. Clin Rehabil 38:932–943. 10.1177/02692155241236609 38425190 10.1177/02692155241236609

[9] Sandroff BM, Silveira SL, Baird JF, Huynh T, Motl RW (2021) Cognitive processing speed impairment does not influence the construct validity of Six-Spot Step Test performance in people with multiple sclerosis. Phys Therapy & J Rehabil 101:1–10. 10.1093/ptj/pzaa227 10.1093/ptj/pzaa227 PMC7910025 33373454

[10] Sandroff BM, Motl RW, Sosnoff JJ, Pula JH (2015) Further validation of the Six-Spot Step Test as a measure of ambulation in multiple sclerosis. Gait Posture 41:222–227. 10.1016/j.gaitpost.2014.10.011 25455207 10.1016/j.gaitpost.2014.10.011

[11] Brincks J, Callesen J, Johnsen E, Dalgas U (2019) A study of the validity of the six-spot step test in ambulatory people with Parkinson’s disease. Clin Rehabil 33:1206–1213 30798635 10.1177/0269215519833016

[12] Brincks J, Callesen J (2021) Examining the test-retest reliability and construct validity of the Six-Spot Step Test in older adults with self-reported balance problems. Clin Rehabil 35:1478–1487. 10.1177/02692155211010278 33874761 10.1177/02692155211010278

[13] Kreutzfeldt M, Jensen HB, Ravnborg M, Markvardsen LH, Andersen H, Sindrup SH (2017) The six-spot-step test - a new method for monitoring walking ability in patients with chronic inflammatory polyneuropathy. J Perip Nerv Sys 22:131–138. 10.1111/jns.12210 10.1111/jns.12210 28407329

[14] Arvidsson Lindvall M, Anderzén-Carlsson A, Appelros P, Forsberg A (2020) Validity and test-retest reliability of the six-spot step test in persons after stroke. Physioth Theory Pra 36:211–218. 10.1080/09593985.2018.1482511 10.1080/09593985.2018.1482511 29873590

[15] Pommerich UM, Brincks J, Skjerbæk AG, Dalgas U (2022) Acta neurologica Belgic 122:893–901. 10.1007/s13760-022-01991-4 10.1007/s13760-022-01991-4 35705789

[16] Grčić PF, Matijaca M, Lušić I, Čapkun V (2011) Responsiveness of walking-based outcome measures after multiple sclerosis relapses following steroid pulses. Medical Science Monitor 17:CR704-CR710. 10.12659/MSM.882130 10.12659/MSM.882130 PMC3628135 22129902

[17] Wallin A, Johansson S, Brincks J, Dalgas U, Franzén E, Callesen J (2024) Effects of balance exercise interventions on balance-related performance in people with multiple sclerosis: a systematic review and a meta-analysis of randomized controlled trials. Neurorehabil Neural Repair 38:775–790. 10.1177/15459683241273402 39162296 10.1177/15459683241273402 PMC11490070

[18] Novotna K, Janatova M, Hana K, Svestkova O, PreiningerovaLizrova J, KubalaHavrdova E (2019) Biofeedback based home balance training can improve balance but not gait in people with multiple sclerosis. Mult Scler Int 2019:2854130. 10.1155/2019/2854130 31934450 10.1155/2019/2854130 PMC6942900

[19] Straudi S, De Marco G, Martinuzzi C, Baroni A, Lamberti N, Brondi L, Da Roit M, Pizzongolo L, Basaglia N, Manfredini F (2022) Combining a supervised and home-based task-oriented circuit training improves walking endurance in patients with multiple sclerosis. The MS_TOCT randomized-controlled trial. Multiple Sclerosis and Related Disorders 60: 103721. 10.1016/j.msard.2022.103721 10.1016/j.msard.2022.103721 35276451

[20] Hoang P, Schoene D, Gandevia S, Smith S, Lord SR (2016) Effects of a home-based step training programme on balance, stepping, cognition and functional performance in people with multiple sclerosis–a randomized controlled trial. J Multi Sclerosis 22:94–103. 10.1177/1352458515579442 10.1177/1352458515579442 25921035

[21] Nilsagård YE, Forsberg AS, von Koch L (2013) Balance exercise for persons with multiple sclerosis using Wii games: a randomised, controlled multi-centre study. J Multi Sclerosis 19:209–216. 10.1177/1352458512450088 10.1177/1352458512450088 22674972

[22] Prosperini L, Fortuna D, Giannì C, Leonardi L, Marchetti MR, Pozzilli C (2013) Home-based balance training using the Wii balance board: a randomized, crossover pilot study in multiple sclerosis. Neurorehabilitaion and Neural Repair 27:516–525. 10.1177/1545968313478484 10.1177/1545968313478484 23478168

[23] Dalgas U, Stenager E, Jakobsen J, Petersen T, Hansen J, Knudsen C, Overgaard K, Ingemann-Hansen T (2009) Resistance training improves muscle strength and functional capacity in multiple sclerosis. Neur 73:1478–1484. 10.1212/WNL.0b013e3181bf98b4 10.1212/WNL.0b013e3181bf98b4 19884575

[24] Mokkink L, Terwee C, de Vet H, Key, (2021) Concepts in clinical epidemiology: Responsiveness, the longitudinal aspect of validity. J Clini Epidem 140:159–162. 10.1016/j.jclinepi.2021.06.002 10.1016/j.jclinepi.2021.06.002 34116141

[25] Kurtzke JF (1983) Rating neurologic impairment in multiple sclerosis: an expanded disability status scale (EDSS). Neur 33:1444–1452. 10.1212/wnl.33.11.144 10.1212/wnl.33.11.1444 6685237

[26] Callesen J, Cattaneo D, Brincks J, KjeldgaardJørgensen ML, Dalgas U (2020) How do resistance training and balance and motor control training affect gait performance and fatigue impact in people with multiple sclerosis? A randomized controlled multi-center study. J Multi Sclerosis 26:1420–1432. 10.1177/1352458519865740 10.1177/1352458519865740 31339460

[27] Callesen J, Cattaneo D, Brincks J, Dalgas U (2018) How does strength training and balance training affect gait and fatigue in patients with Multiple Sclerosis? A study protocol of a randomized controlled trial. Neuro Rehabil 42:131–142. 10.3233/NRE-172238 10.3233/NRE-172238 29562556

[28] Prinsen CAC, Mokkink LB, Bouter LM, Alonso J, Patrick DL, de Vet HVW, Terwee CB (2018) COSMIN guideline for systematic reviews of patient-reported outcome measures. Qual Life Res 27:1147–1157. 10.1007/s11136-018-1798-3 29435801 10.1007/s11136-018-1798-3 PMC5891568

[29] Nilsagård YE, von Koch LK, Nilsson M, Forsberg AS (2014) Balance exercise program reduced falls in people with multiple sclerosis: a single-group, pretest-posttest trial. Arc of Phys Med Rehabil 95:2428–2434. 10.1016/j.apmr.2014.06.016 10.1016/j.apmr.2014.06.016 25004466

[30] Cattaneo D, Jonsdottir J, Zocchi M, Regola A (2007) Effects of balance exercises on people with multiple sclerosis: a pilot study. Clin Rehabil 21:771–781. 10.1177/0269215507077602 17875557 10.1177/0269215507077602

[31] Halvarsson A, Dohrn IM, Ståhle A (2015) Taking balance training for older adults one step further: the rationale for and a description of a proven balance training programme. Clin Rehabil 29:417–425. 10.1177/0269215514546770 25200877 10.1177/0269215514546770 PMC4419050

[32] Hebert JR, Corboy JR, Manago MM, Schenkman M (2011) Effects of vestibular rehabilitation on multiple sclerosis-related fatigue and upright postural control: a randomized controlled trial. J Phys Therapy & Rehabil 91:1166–1183. 10.2522/ptj.20100399 10.2522/ptj.20100399 21680771

[33] Fischer JS, Rudick RA, Cutter GR, Reingold SC (1999) The Multiple Sclerosis Functional Composite Measure (MSFC): an integrated approach to MS clinical outcome assessment. National MS Society Clinical Outcomes Assessment Task Force. J Multi Sclerosis 5:244–250. 10.1177/135245859900500409 10.1177/135245859900500409 10467383

[34] COSMIN (2019) Study Design checklist for Patient-reported outcome measurement instruments. COSMIN. https://www.cosmin.nl/tools/checklists-assessing-methodological-study-qualities/. Accessed 16 June 2025

[35] Meseguer-Henarejos AB, López-Pina JA, López-García JJ, Martínez-González-Moro I (2025) Psychometric properties of the Mini-Balance Evaluation Systems Test (Mini-BESTest) among multiple populations: a COSMIN systematic review and meta-analysis. Disabil Rehabil. 10.1080/09638288.2025.2456602 39873412 10.1080/09638288.2025.2456602

[36] Wallén MB, Sorjonen K, Löfgren N, Franzén E (2016) Structural Validity of the Mini-Balance Evaluation Systems Test (Mini-BESTest) in People With Mild to Moderate Parkinson’s Disease. J Phys Therapy & Rehabil 96:1799–1806. 10.2522/ptj.20150334 10.2522/ptj.20150334 27231272

